# Topology-dependent asymmetry in systematic errors affects phylogenetic placement of Ctenophora and Xenacoelomorpha

**DOI:** 10.1126/sciadv.abc5162

**Published:** 2020-12-11

**Authors:** Paschalia Kapli, Maximilian J. Telford

**Affiliations:** Centre for Life’s Origins and Evolution, Department of Genetics, Evolution and Environment, University College London, Gower Street, London WC1E 6BT, UK.

## Abstract

The evolutionary relationships of two animal phyla, Ctenophora and Xenacoelomorpha, have proved highly contentious. Ctenophora have been proposed as the most distant relatives of all other animals (Ctenophora-first rather than the traditional Porifera-first). Xenacoelomorpha may be primitively simple relatives of all other bilaterally symmetrical animals (Nephrozoa) or simplified relatives of echinoderms and hemichordates (Xenambulacraria). In both cases, one of the alternative topologies must be a result of errors in tree reconstruction. Here, using empirical data and simulations, we show that the Ctenophora-first and Nephrozoa topologies (but not Porifera-first and Ambulacraria topologies) are strongly supported by analyses affected by systematic errors. Accommodating this finding suggests that empirical studies supporting Ctenophora-first and Nephrozoa trees are likely to be explained by systematic error. This would imply that the alternative Porifera-first and Xenambulacraria topologies, which are supported by analyses designed to minimize systematic error, are the most credible current alternatives.

## Teaser

A data simulation approach shows that the contested origins of Ctenophora and Xenacoelomorpha are obscured by systematic errors.

## INTRODUCTION

Knowing the relationships between major groups of animals is essential for understanding the earliest events in animal evolution. While the use of molecular data has led to considerable progress in understanding animal relationships, some aspects remain highly disputed. The positions of two animal phyla, Ctenophora (sea gooseberries) and Xenacoelomorpha (simple marine worms including xenoturbellids and acoelomorph worms), have proved particularly contentious. Ctenophora have been proposed as the most distant relatives of all other animals (Ctenophora-first topology), although they have muscles, nerves, and other characters absent in sponges (classically the sister group of other animals: Porifera-first topology). Xenacoelomorphs may be the sister group of all other bilaterians (they are simple and lack characteristics of other Bilateria: Nephrozoa topology); alternatively, they have been linked to the complex Ambulacraria (Hemichordata and Echinodermata: Xenambulacraria topology), implying loss of complexity.

**Fig. 1:**
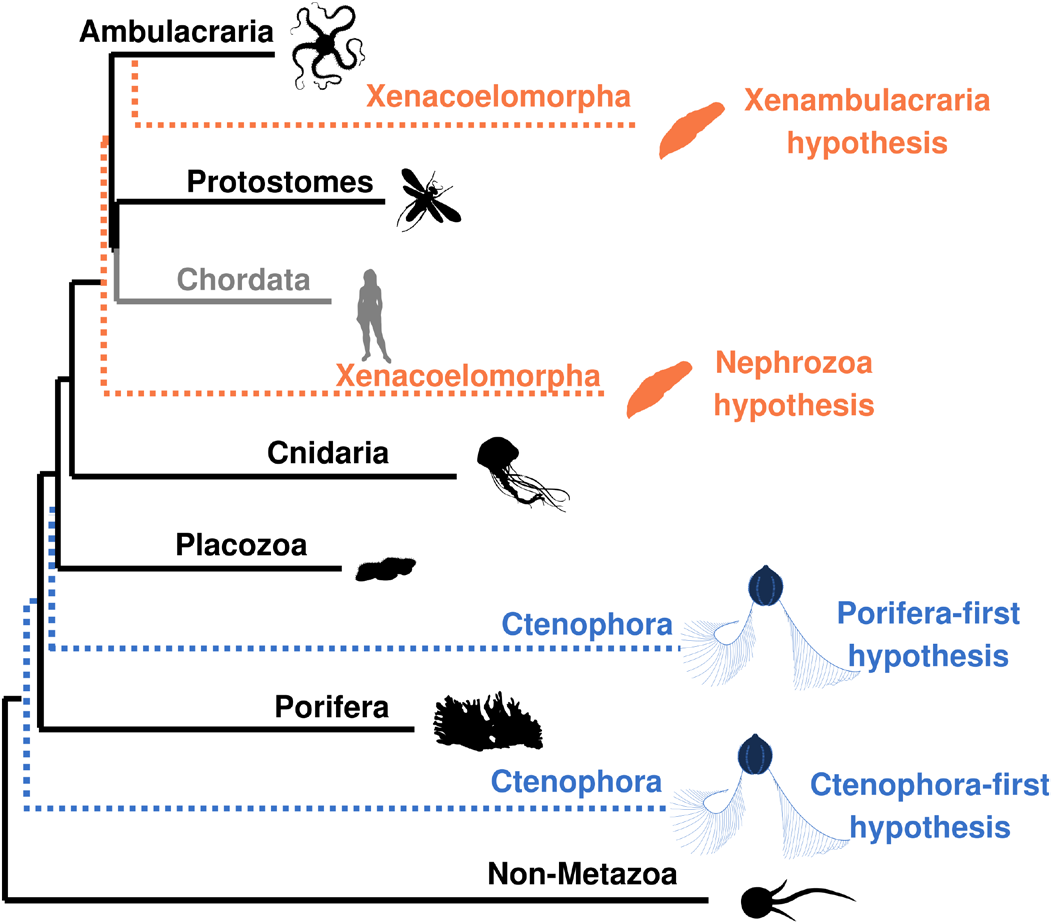
Phylogenetic tree of main animal groups highlighting alternative hypotheses for the positions of the Ctenophora and Xenacoelomorpha. The dotted lines show alternative positions for the Ctenophora and Xenacoelomorpha. The sister group of all other Metazoa could be Ctenophora (Ctenophora-first) or the Porifera (Porifera-first). Xenacoelomorpha could be the sister group of the Ambulacraria (Xenambulacraria hypothesis), or the Xenacoelomorpha could be the sister group of all other Bilateria (Nephrozoa hypothesis). Branch lengths are approximately proportional to the average branch lengths leading to the clades indicated. Long branches leading to Ctenophora and Xenacoelomorpha are evident. The Chordata are shown as a sister group of the Protostomia: a topology supported by the dataset used in our analyses (*9*).

While the Ctenophora-first and the Nephrozoa topologies (Fig. 1) have gained support in independent analyses of large datasets (*1*–*6*), supporters of the alternative topologies (Porifera-first and Xenambulacraria; Fig. 1) have suggested that there is an expectation of a long-branch attraction (LBA) artifact (*7*–*9*). LBA is a systematic error that falsely groups long branches (*10*), such as those leading to the outgroups and to both the Ctenophora and Xenacoelomorpha. LBA can be exacerbated by the use of substitution models that do not account for heterogeneities in sequence evolution such as nonhomogeneous rates of substitution between alignment sites or heterogeneities in the frequencies of amino acids across the alignment (*11*–*14*). Attraction between the long branches leading to the Ctenophora and Xenacoelomorpha and to their respective outgroups could result in the Ctenophora-first and Nephrozoa trees. The support seen for Ctenophora-first and Nephrozoa topologies, however, is generally stronger than for the alternatives (*8*, *9*).

We have used recently published phylogenomic datasets that were designed to place either the ctenophores [Simion *et al.* (*8*); dataset “Simion-all”] or the xenacoelomorphs [Philippe *et al.* (*9*) and Cannon *et al.* (*5*); datasets “Philippe-all” and “Cannon”] in the animal tree. We ask whether unaccounted-for across-site heterogeneity in amino acid composition might have resulted in model violations that could lead to the underestimation of the prevalence of convergent evolution. We then ask whether ignoring such heterogeneity could result in LBA and incorrect support for the Ctenophora-first and Nephrozoa trees.

## RESULTS

### Effect of model misspecification on accuracy of branch length estimation

**Fig. 2:**
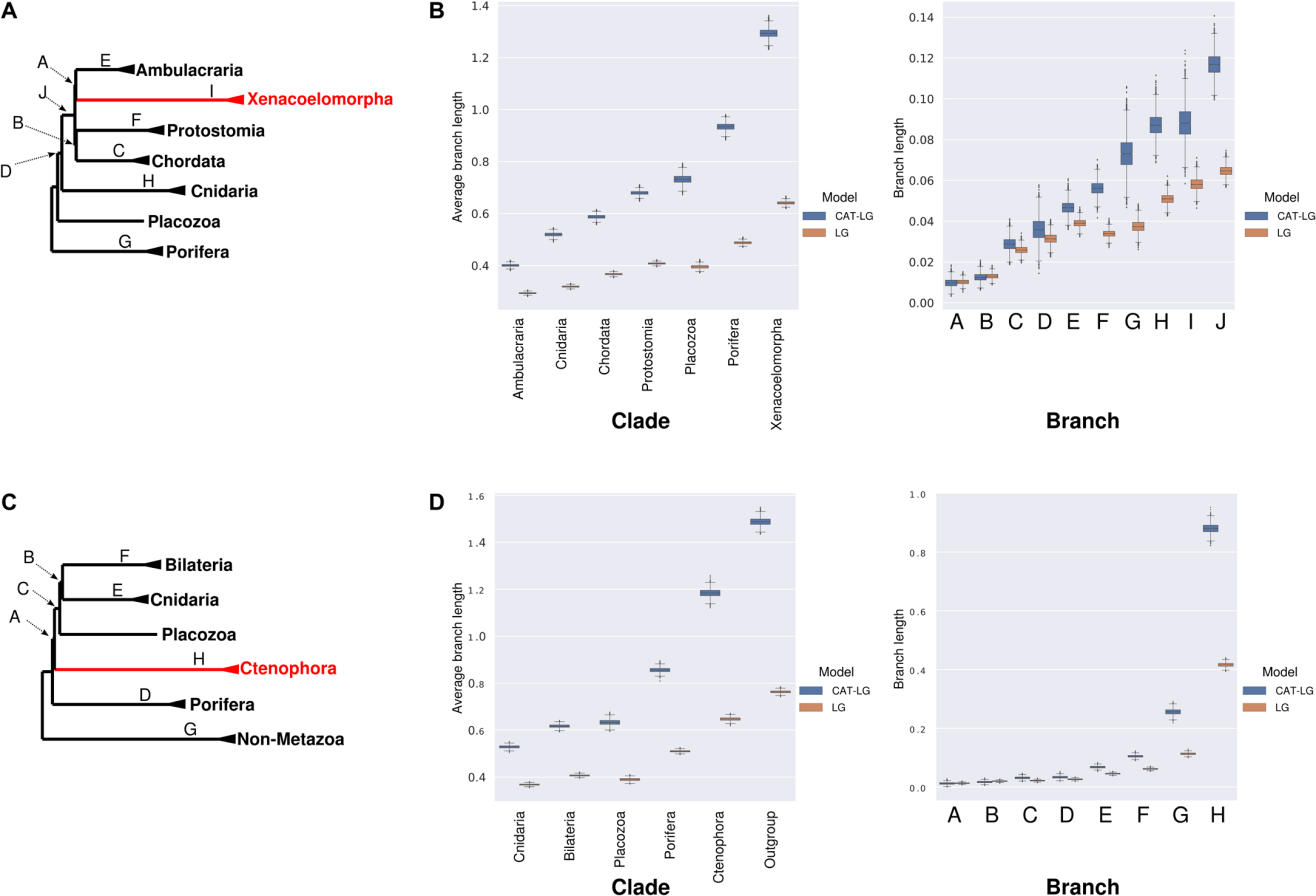
Site-homogeneous models consistently underestimate branch lengths. (**A**) Tree showing the clades (names) and branches (letters) for which lengths were estimated using Philippe-all data. (**B**) Estimates of clade and branch lengths using site-heterogeneous model (blue) and site-homogeneous model (brown) based on empirical data. (**C**) Tree showing the clades and branches for which lengths were estimated using Simion-all data. (**D**) Estimates of clade and branch lengths using site-heterogeneous (blue) model and site-homogeneous model (brown) based on empirical data. Site-homogeneous models consistently estimate shorter branch lengths.

We first used Bayesian inference (*15*) to assess the difference in branch length estimates under site-heterogeneous (i.e., CAT + LG + G) and site-homogeneous (i.e., LG + G) models. We used a fixed topology to reduce the computational burden and performed the calculations on a subset of the original data (30,000 sites). We observe that branch lengths estimated under a site-homogeneous model are consistently shorter than those estimated under a site-heterogeneous model for both Simion-all and Philippe-all datasets. Notably, the discrepancy between branch lengths estimated using homogeneous and heterogeneous models was proportionally larger for longer branches (Fig. 2).

**Fig. 3:**
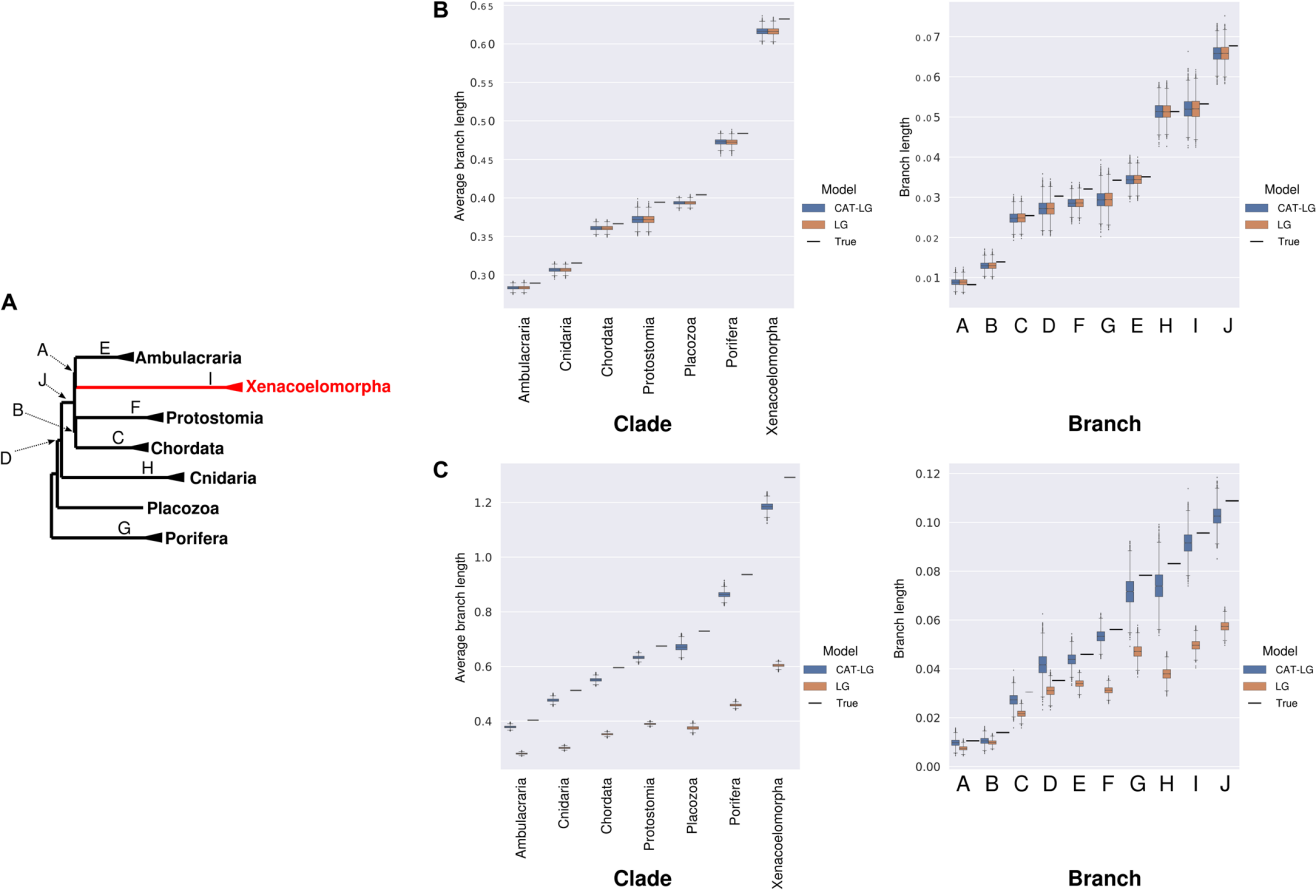
Site-heterogeneous models estimate accurate branch lengths for both site-homogeneous and site-heterogeneous data. (**A**) The guide tree that was used for simulating the data, showing the clades (names) and branches (letters) used for comparing the estimates across models. (**B**) Estimates of clade and branch lengths for data simulated under the site-homogeneous model and inferred using the site-heterogeneous model (blue) and site-homogeneous model (brown). Both models give similarly accurate branch lengths for data simulated with the site-homogeneous model; the true branch lengths are shown with the black lines. (**C**) Equivalent estimates of clade and branch lengths for data simulated under the site-heterogeneous model. Site-heterogeneous models give accurate estimates, whereas site-homogeneous models consistently underestimate the amount of change/branch length.

It is possible that site-homogeneous models have underestimated or that site-heterogenous models overestimated branch lengths (or both could be true). To evaluate which of these possibilities is responsible for the observed differences, we simulated amino acid sequences using empirically calibrated parameter values (i.e., values estimated from empirical data) under both site-homogeneous and site-heterogeneous models. We assessed the efficiency of both model types in estimating the known branch lengths correctly. Both models give accurate estimates of branch lengths for data that have evolved homogeneously, showing that site-heterogenous models do not systematically overestimate branch lengths. For data that have evolved heterogeneously, however, site-homogeneous models consistently underestimate branch lengths (Fig. 3). This result shows that using site-homogeneous models will consistently underestimate branch lengths for data that have evolved heterogeneously. The discrepancy between the branch length estimates using the two models for site-heterogeneous data resembles the discrepancy that we observe for the empirical data (Fig. 2). The need to accommodate site heterogeneity is also supported by cross-validation tests [this study and see (*9*)], which show that a site-heterogeneous model fits these data significantly better than a site-homogeneous model (Fig. 3).

### Effect of model misspecification on topology

While the branch lengths in both datasets are shown to be consistently underestimated under a homogeneous model, it need not follow that this will affect our ability to reconstruct the tree topology correctly. To see the effect of model violations on topology, we simulated data (100 replicates) using a site-heterogeneous model under each of the two alternative topologies for each dataset (i.e., Nephrozoa/Xenambulacraria and Ctenophora-first/Porifera-first). We performed the simulations with PhyloBayes (*15*) using parameters estimated under the site-heterogeneous model and the relevant topological hypothesis. For each simulated dataset, we inferred two maximum likelihood phylogenies using IQ-TREE (*16*): (i) under an empirical site-heterogeneous model (C60 + LG + G) that serves as an approximation of the CAT model ((*17*) and (ii) under a site-homogeneous model (LG + G). We also performed bootstrap analyses using the site-homogeneous model (LG + G) and the more complex site-heterogeneous model (C60 + LG + G) to assess the robustness of the inferred phylogenies with respect to the nodes of interest.

**Fig. 4:**
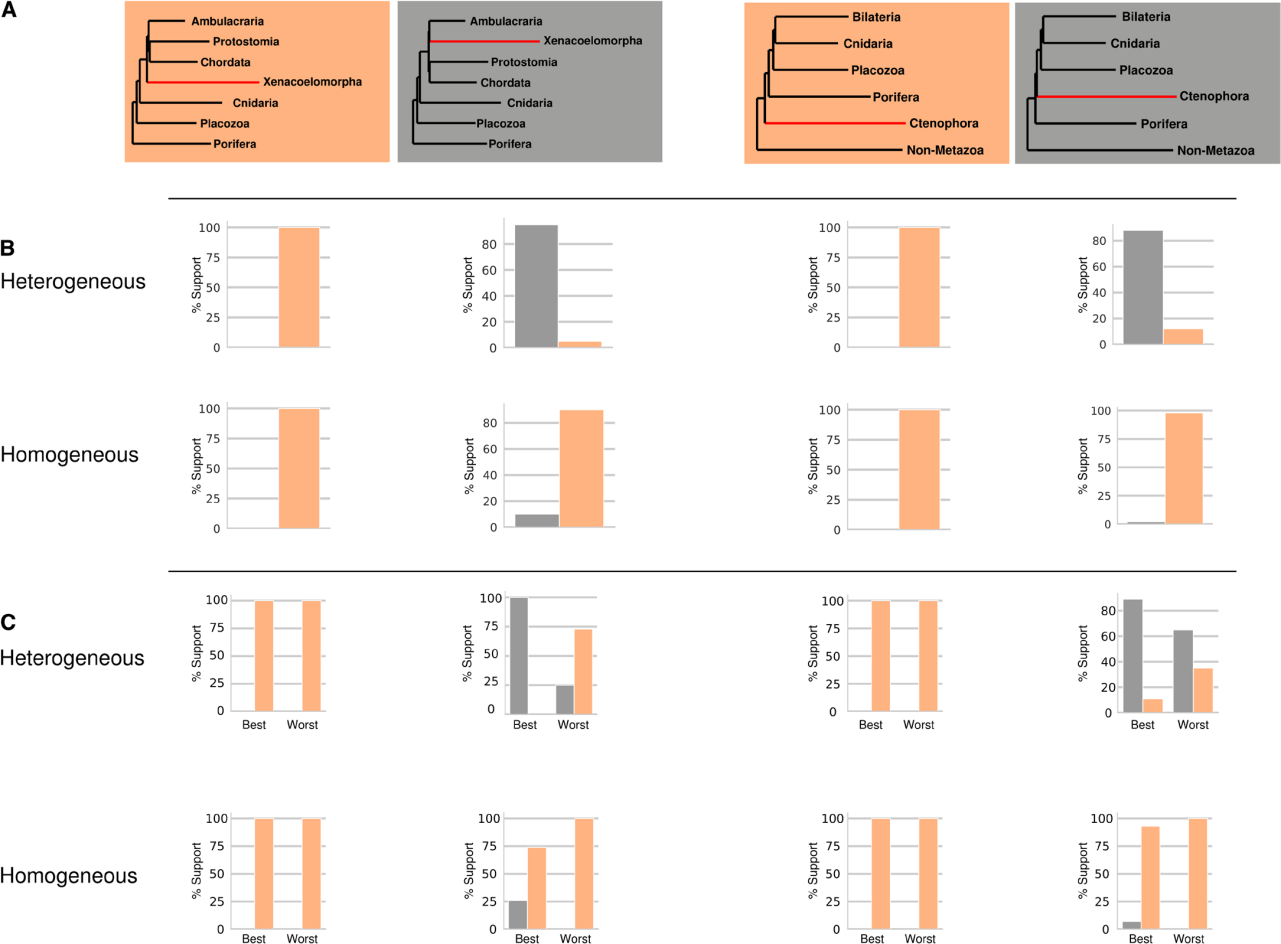
Topology-dependent asymmetry of the ability of model-violating site-homogeneous models to reconstruct the correct tree. (**A**) A total of 100 datasets were simulated using a site-heterogeneous model for each of the topologies shown (orange/gray boxes). (**B**) For the datasets based on the whole alignment, site-heterogeneous (top) and site-homogeneous models (bottom) were used to reconstruct a maximum likelihood tree. The proportion of times the orange or black tree was reconstructed is shown in the bar charts. Data simulated under the Nephrozoa and the Ctenophora-first trees always yield the correct topology regardless of the model. Data simulated under the Xenambulacraria and Porifera-first topologies mostly yield the correct topology under the site-heterogeneous model but an incorrect topology under the site-homogeneous model. The incorrect tree is always Nephrozoa and the Ctenophora-first, respectively. (**C**) The experiments were repeated for the datasets based on the sets of genes best and worst at reconstructing known clades. For the best genes under both models, the inference is improved for data simulated under Xenambulacraria and Porifera-first topologies. A decrease in the performance of both models is observed using the worst data.

**Fig. 5:**
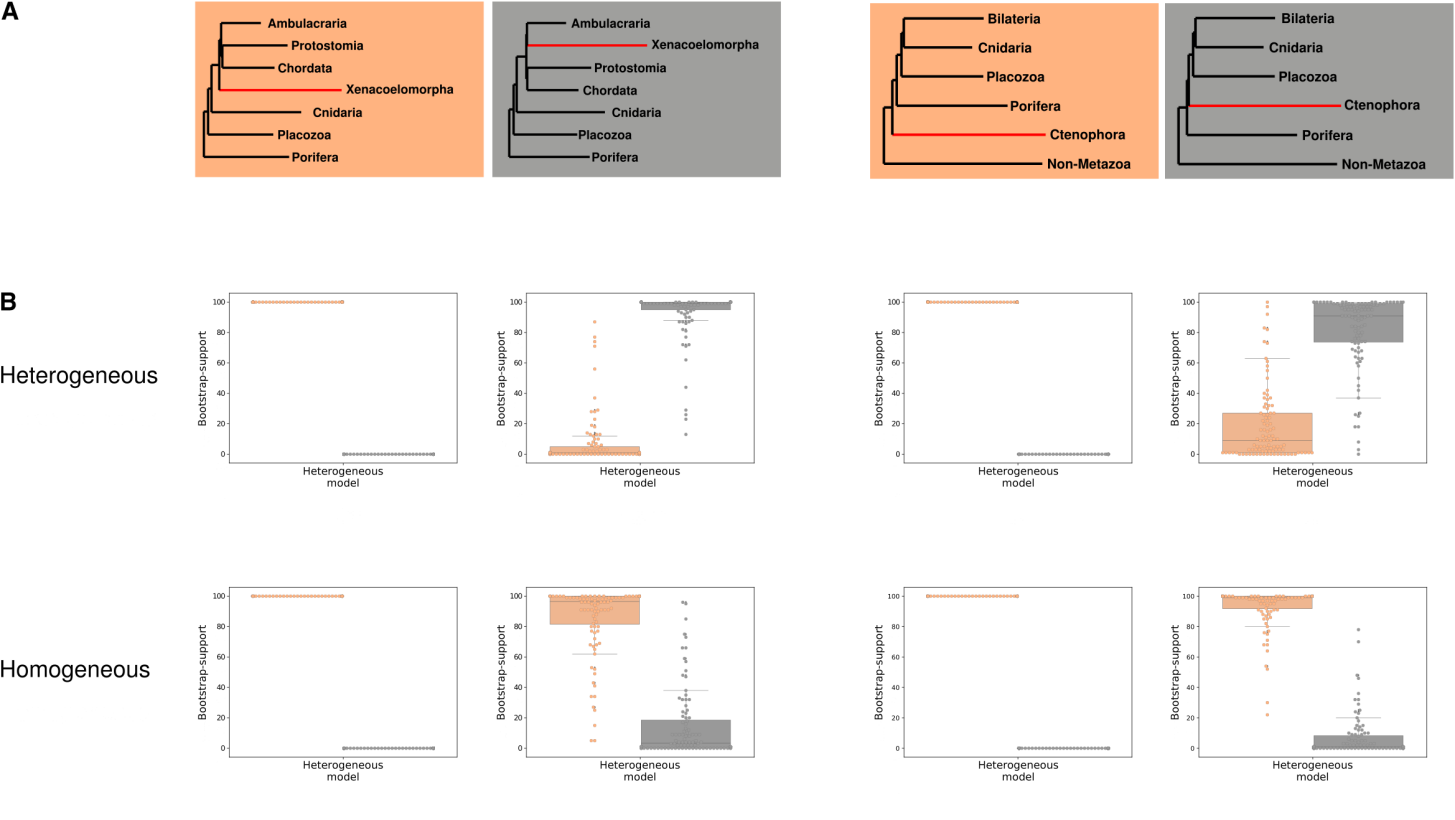
Topology-dependent asymmetry of tree reconstruction analyses shown using bootstrap. (**A**) A total of 100 datasets were simulated using a site-heterogeneous model for each of topologies shown in the corresponding box plots (orange/gray boxes). (**B**) Site-heterogeneous (top) and site-homogeneous models (bottom) were used to reconstruct a maximum likelihood tree, with the bootstrap support measured. The bootstrap support values showing the support of either gray or orange topologies are shown in the bar charts. Data simulated under the Nephrozoa and the Ctenophora-first trees always yield the correct topology regardless of the model with 100% bootstrap support. Data simulated under the Xenambulacraria and Porifera-first topologies mostly yield the correct topology under the site-heterogeneous model but an incorrect topology under the site-homogeneous model. The incorrect tree is always Nephrozoa or Ctenophora-first, respectively.

For datasets simulated under the potential LBA topologies (Nephrozoa and Ctenophora-first), we find that the correct tree was reconstructed in 100% of cases even under model violation (Fig. 4) and with 100% bootstrap support (Fig. 5). In contrast, the data simulated under the alternative Xenambulacraria and Porifera-first topologies yielded incorrect topologies under the site-homogeneous model (in 90 and 98% of simulations, respectively). In all cases, the incorrect topology was the putative LBA topology (Fig. 4 and table S1), and this topology was almost always supported by high bootstrap values (Fig. 5). The same data analyzed under the site-heterogeneous C60 + LG + G model recovered the correct tree 95% of the time for Xenambulacraria and 88% of the time for Porifera-first, with high bootstrap support (Fig. 5).

This marked asymmetry of the effects of model misspecification on our ability to recover the two topologies implies that, using these data, we are highly unlikely to reconstruct the Porifera-first or Xenambulacraria trees in error if the alternative topologies are true. We show, however, that model misspecification leading to branch length underestimation is highly likely to result in a failure to reconstruct the Porifera-first or Xenambulacraria topologies correctly. We repeated these experiments constraining the deuterostomes to be monophyletic [a topology not supported by the Philippe data (*9*)] as well as using the Cannon data (*5*), which do support monophyletic deuterostomes. While Xenambulacraria is more easily recovered under these conditions (the alternative Nephrozoa is less easily recovered in error), we nevertheless find that for data simulated under the Nephrozoa topology with monophyletic deuterostomes, we never recover the Xenambulacraria tree in error (table S1).

For data simulated under a homogeneous model, all four topologies were always correctly reconstructed using either model. If the state frequencies were homogeneous across the alignment, we should expect no errors in phylogenetic inference regardless of the true topology (table S1).

### Measuring the degree of asymmetry

To measure the asymmetry in the ease with which the Nephrozoa and Xenambulacraria topologies can be reconstructed, we made datasets composed of different proportions of data simulated under the two topologies. If there were no asymmetry, datasets composed of 50% from each simulation should support each topology ~50% of the time. We find instead that reconstructing trees based on balanced (50/50) datasets and using the site-heterogeneous models result in support for Nephrozoa 100% of the time. Only when we increase the proportion of data coming from simulations based on the Xenambulacraria tree to reach 90% Xenambulacraria versus 10% Nephrozoa does support for Xenambulacraria outweigh support for Nephrozoa (table S2). The bias favoring Nephrozoa is even stronger for datasets constraining the deuterostomes to be monophyletic (table S2).

### Effect of long terminal branches on topology

Long terminal branches and unaccommodated heterogeneities are important conditions contributing to the existence of LBA. To investigate the impact of long branches in the Xenacoelomorpha example, we repeated our simulation experiment having first removed the long-branched Acoelomorpha, leaving just the relatively short-branched *Xenoturbella*. This is common practice for reducing LBA artifacts in empirical studies (*18*–*20*). All sampled ctenophores are found at the end of a long branch, meaning that this taxon trimming experiment could not be done in this case. Consistent with the importance of a contribution from extreme long branches to the LBA error, once this had been reduced by removing acoelomorphs, the Xenambulacraria topology was recovered for 52% of the simulated alignments even under model violation. Under the site-heterogeneous model, the correct topology was recovered more frequently (89%), although two of the simulated datasets yielded an alternative erroneous placement of Xenacoelomorpha, i.e., as sister to Protostomia and Chordata. In simulations using the Nephrozoa topology, removing the long-branched acoelomorphs had no effect on our ability to reconstruct the correct tree using either model. Overall, this experiment suggests that, if the Xenambulacraria tree is correct, then the observed long branches leading to the Acoelomorpha would contribute to artifactual support for the Nephrozoa tree and this recapitulates findings with empirical data (table S1) (*9*).

### Effect of short internal branches on topology

Other conditions can also exacerbate the phenomenon of LBA, which is expected to be more prevalent for trees with few informative changes along the internal branches separating the clades of interest (short internal branches). We tested this by examining the ability of subsets of genes containing shorter/longer internal branches to resist LBA artifacts.

**Fig. 6:**
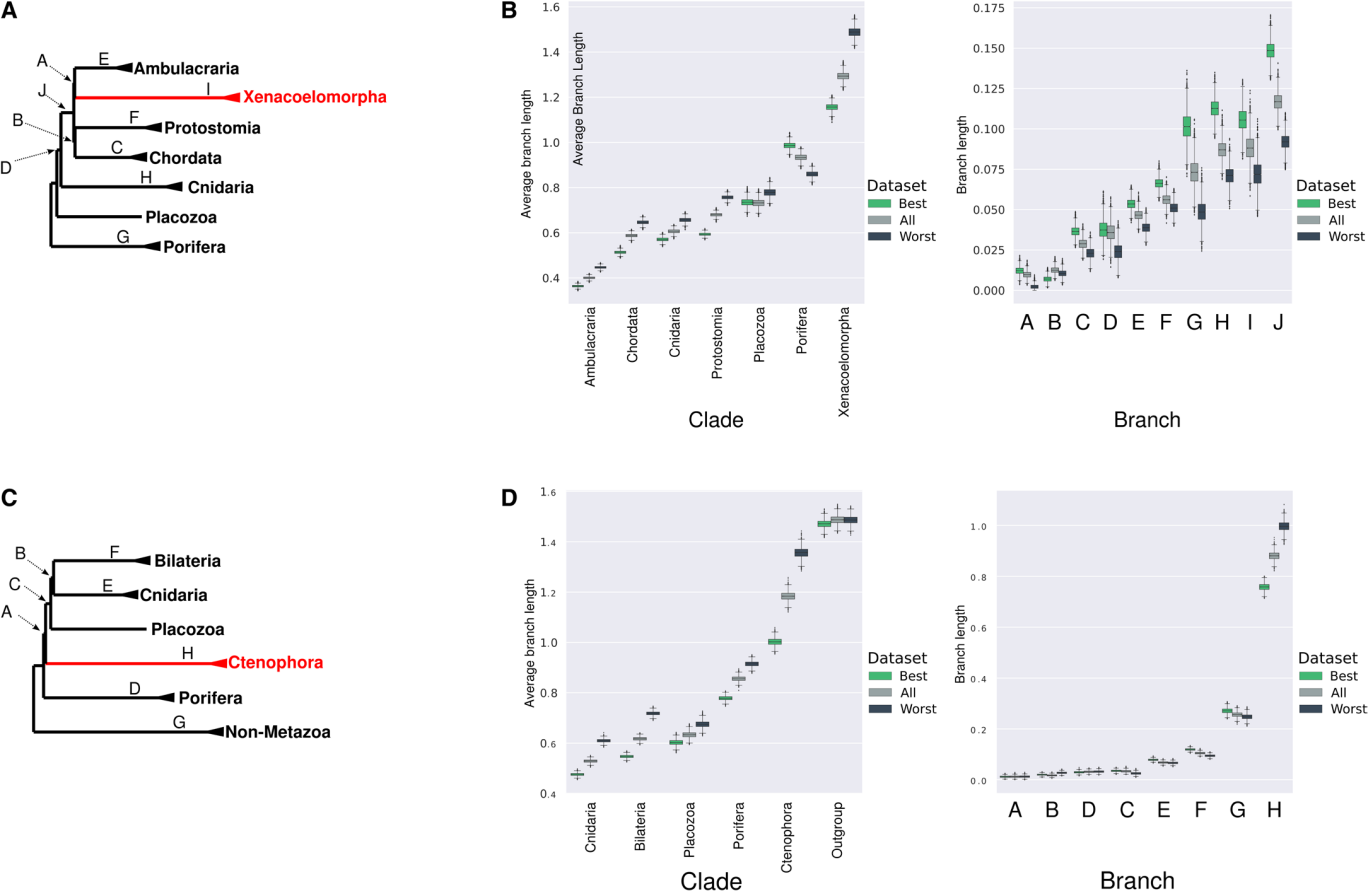
Best genes have short terminal branches and longer internal branches. (**A**) A tree showing the clades (names) and branches (letters) for which lengths were estimated for the Philippe data. (**B**) Estimates of clade and branch lengths for empirical data using a site-heterogeneous model for three data samples: best genes (green, highest monophyly scores), all dataset (gray), and worst genes (black, lowest monophyly scores). Best genes have shorter terminal branches within clades than all or worst. Best genes have longer branches separating clades than all or worst. (**C**) A tree showing the clades (names) and branches (letters) for which lengths were estimated for the Simion data. (**D**) Estimates of clade and branch lengths for the Simion-best, Simion-all and Simion-worst genes. Best genes have shorter terminal branches within clades than all or worst. For the best genes, most internal branches are the same or longer than for all or worst genes with the exception of the internal branch leading to the Ctenophora clade.

Philippe *et al.* ranked their 1173 genes from those most able to reconstruct known clades (“best”) to those least able (“worst”) on the basis of the ability of each gene to reconstruct known clades as monophyletic (monophyly score). We made estimates of terminal and internal branch lengths from 30,000 alignment positions randomly drawn from the sets of 25% best and 25% worst genes and compare these to the estimates that we have described for 30,000 positions drawn randomly from the whole alignment. We show that the reduction in tree length in the best quarter of genes (*9*) comes from a reduction of the terminal branches but not of the internal. We find that the internal branches are longer when estimated using the best genes than when using the whole dataset or the worst genes (Fig. 6.). The situation observed in the best genes describes the ideal situation if we wish to lessen LBA (*12*, *21*, *22*).

To test the effects of this gene selection on our ability to infer the correct topology, we simulated site-heterogeneous data under the Xenambulacraria and Porifera-first trees using parameters derived from the worst and best genes. Trees reconstructed from the worst data using the site-heterogeneous C60 + LG + G model show a clear reduction in support for the correct tree compared to the total dataset (Xenambulacraria: all genes, 95% correct and worst, 25% correct; Porifera-first: all genes, 88% correct and worst, 65% correct). For these worst data, we also occasionally recovered Xenacoelomorpha as sister to Protostomia and Chordata (in 2% of the simulated datasets consisting of all taxa and in 5% of the simulated datasets without acoels). For datasets simulated using parameters estimated from the best genes, the correct tree is reconstructed 100% of the time (Xenambulacraria) and 89% of the time (Porifera-first). However, even under the site-homogeneous model, the correct tree is reconstructed 26% of the time (Xenambulacraria) and 7% of the time (Porifera-first). When long-branched Acoelomorpha are removed, the correct Xenambulacraria topology is reconstructed 92% of the time for simulated data based on the best genes even with a site-homogeneous model.

In contrast to these results, using data simulated according to the putative LBA topologies (Nephrozoa and Ctenophora-first), the correct topology is always recovered regardless of the dataset or model used (Fig. 4). The Ctenophora-first/Nephrozoa topologies are trivial to reconstruct correctly even with poor data or under model violation.

### Accuracy correlates with the complexity of site-heterogeneous models

To test the effects of models of intermediate complexity, we repeated our analyses using a second site-heterogeneous model with fewer site frequency profiles (C10 + LG + G) on data simulated using a site-heterogeneous model under the Xenacoelomorpha hypothesis. We found that, using this model that has a complexity (and fit) intermediate between the site-homogeneous models and the relatively complex C60 model, we recovered intermediate results for data simulated under the Porifera-first and Xenambulacraria hypotheses for all datasets (best, all, and worst). Trees were reconstructed correctly more often than when using site-homogeneous models but less often than with C60 (table S1). For the data simulated under the alternative Ctenophora-first and Nephrozoa hypotheses, the correct topology was recovered in all cases.

Building on this, we repeated the experiment for a subset of datasets using the more complex CAT + LG + G model using the PhyloBayes software. For all datasets simulated under the Nephrozoa (10 datasets) or Ctenophora-first topologies (10 datasets), we reconstructed the correct tree. For datasets simulated under the Xenambulacraria or Porifera-first topologies, we considered 10 datasets that C60 correctly reconstructed and 10 for which C60 incorrectly reconstructed the Ctenophora-first and Nephrozoa trees, respectively. For the former, the CAT + LG + G model also always reconstructed the correct tree with posterior probability (pp) = 1.0. For the latter, the CAT + LG + G model succeeded where the C60 model had failed albeit with pp <1 in some replicates, and in two datasets, when using CAT, the Xenacoelomorpha were recovered as sister to Protostomes and Chordates. Empirical data may suffer from additional heterogeneous processes (*23*, *24*) not captured by the CAT model and therefore also absent in our simulations. Failing to adequately model any such additional heterogeneity can have a similar effect to failing to model site frequency heterogeneity. It should be expected, therefore, that in empirical studies, even the CAT model may fail to overcome LBA artifacts. These experiments suggest, nevertheless, that better fitting models are better able to overcome these LBA artefacts.

Last, we wanted to examine concerns (*25*) that the support for the Porifera-first and Xenambulacraria trees that has been observed when analyzing empirical data using the CAT-F81 model may be the result of an error stemming from the radical assumptions about amino acid exchangeabilities in CAT-F81. We considered 10 datasets simulated using a site-heterogeneous model under each of the Ctenophora-first and Nephrozoa topologies and reanalyzed these using the CAT-F81 model. We find that, with these data, the use of the CAT-F81 model always gave the correct tree, never resulting in incorrect support for Porifera-first or for Xenambulacraria.

## DISCUSSION

There have already been many analyses attempting to resolve the positions of the Ctenophora and Xenacoelomorpha (*1*–*9*, *26*, *27*). These have used increasingly large datasets and various complex analyses and data filtering schemes. In each of the two cases, there are two entrenched camps and little apparent progress. Implicit in the recent arguments in the scientific literature is the idea that there is a fine balance between the two proposed positions (*28*). One implication of this is that it will take even more data or more sophisticated analysis to tease out some elusive signal and to nudge opinion in one direction or another. What we find, in contrast, is that there is a major and so far underappreciated asymmetry between the two possible solutions. They are not equivalent, and our results show that the resulting change to sensible prior expectations should have a major influence on interpretation of published results.

Supporters of the Porifera-first and Xenambulacraria topologies have long suggested that the alternatives, Ctenophora-first and Nephrozoa, result from an LBA artifact. The long branches leading to the Xenacoelomorpha and Ctenophora and the short branches relating them to other phyla suggested that their placement might be particularly susceptible to systematic errors. Our simulations, using realistic parameters drawn from empirical data, show just how important this artifact is.

Using realistic simulations under the Ctenophora-first and Nephrozoa topologies, we show that we never recover the alternatives (Porifera-first and Xenambulacraria) in error. We show that Ctenophora-first and Nephrozoa trees, if they were true, would gain exaggerated artifactual support due to the effects of LBA. This effect is well known as the “Farris zone” (*29*) or “inverse Felsenstein zone” (*30*): the artificial reinforcement of a close relationship between two long branches by LBA. If the Ctenophora-first and Nephrozoa trees were true, then there is a very low likelihood that the support that empirical studies have shown for the alternatives would ever be observed (*14*).

In contrast, the Porifera-first and Xenambulacraria trees are highly susceptible to LBA effects. We have shown that the Xenambulacraria and Porifera-first trees are both strongly affected by the long terminal branches leading to the respective phyla, by short internal branches, and by unaccounted for site heterogeneity, making these trees difficult to recover. Under conditions that emphasize LBA, we very frequently recover the wrong topologies. The wrong topologies that we observe are the Ctenophora-first and Nephrozoa trees in almost every instance. More generally, our simulations based on Porifera-first and Xenambulacraria topologies accurately predict exactly the effects of long branches and inadequate models that have been observed using real data.

Our findings suggest that the Ctenophora-first and Nephrozoa trees are plausibly interpreted as artifacts. Support seen in empirical studies for the alternative Porifera-first and Xenambulacraria trees are unlikely to be artefacts, and the implication is that these trees are likely to be correct. This would suggest that the most plausible sister group of all other animals is the Porifera and not the Ctenophora and that the Xenacoelomorpha is likely to be the sister group of the Ambulacraria and not a branch intermediate between Cnidaria and the rest of the Bilateria.

## MATERIALS AND METHODS

### Experimental design

To test the effects of model misspecification on our ability to reconstruct the position of the Xenacoelomorpha and Ctenophora correctly, we used empirical and simulated data. We first used empirical data to assess the effects of site-heterogeneous and site-homogeneous substitution models on branch length estimation.

We then simulated data under the two models and the conflicting topologies based on parameters learned from the empirical data. On the basis of the simulated data, we evaluated the performance of site-homogeneous and site-heterogeneous models in branch length estimation for both site-homogeneous and site-heterogeneous data.

We used the simulated data to estimate tree topologies using different models to see the effect of model misspecification on our ability to reconstruct the correct tree. We used different data samples (removing certain species or genes) to see the effects of this on our ability to reconstruct the correct tree.

### Data

For the majority of the analyses, we used two recently published phylogenomic datasets: (i) one focusing on the placement of Ctenophora [“Simion” (*8*)], which comprises 97 taxa of which 72 are metazoans and 25 are nonmetazoans, and (ii) a dataset aimed at resolving the placement of Xenacoelomorpha [“Philippe” (*9*)] consisting of 59 taxa of which 45 are bilaterians and 14 are outgroups.

Both datasets had been filtered for potential contaminants, paralogs, and other outlier sequences (more details are provided in the original papers). Following this filtering, the two datasets consist of 401,632 (Simion) and 353,607 (Philippe) amino acid positions. In both cases, these datasets are impractically large for repeated phylogenetic inference, particularly in a Bayesian inference framework. To ease the computational burden, we randomly selected, from each complete dataset, a subset of 30,000 amino acid positions for the downstream analyses. We refer to the two resulting datasets as Simion-all and Philippe-all.

To study the effect of different gene sets on the placement of Xenacoelomorpha and Ctenophora, we also created two additional subsets of 30,000 randomly selected positions per dataset. For both sets of genes (Simion and Philippe), we scored each individual gene on the basis of its ability to reconstruct uncontested clades [sensu (*9*)]. We then ranked the genes on the basis of this monophyly score and concatenated genes according to their rank from best to worst. For the Simion *et al.* data, we considered as uncontested clades the Homoscleromorpha, Calcarea, Hexactinellida, Demospongiae, Ctenophora, Bilateria, Medusozoa, Anthozoa, and Metazoa. For the Phillipe *et al.* data, we assumed the same groups as in the original paper (*9*). After concatenating the genes from best scoring to worst scoring, we randomly sampled 30,000 alignment sites from the first quarter of the alignment, i.e., the highest scoring (“Simion-best” and “Philippe-best”), and 30,000 from the final quarter of the alignment, i.e., the lowest scoring (“Simion-worst” and “Philippe-worst”). Last, we examined the placement of Xenacoelomorpha with respect to two more factors: (i) the exclusion of the fast evolving Acoelomorpha and (ii) the monophyly of Deuterostomia that represents the traditional view of the relationships relating the Chordata and Ambulacraria.

For the first case, we removed all acoelomorph taxa from the Philippe data and kept only slowly evolving xenoturbellid. After removing these taxa, we performed the same subsampling as before, i.e., we randomly sampled 30,000 amino acids from the entire alignment (“Philippe-no_acoels-all”), the best-scoring genes (“Philippe-no_acoels-best”), and the worst-scoring genes (“Philippe-no_acoels-worst”) as before.

Overall, we considered eight topologies summarized in fig. S1. Two of these reflect the conflicting placement of Ctenophora (topologies A and B in fig. S1). For both of these trees, the rest of the phylogeny was the same as the one published in (*8*) (their “tree_97sp_CAT.tre”). For the placement of Xenacoelomorpha, there are six alternative topologies (C to H in fig. S1), for which the phylum is placed as sister either to Nephrozoa or to Ambulacraria. In four of them, we assumed that deuterostomes are paraphyletic, either with all the Xenacoelomorpha included (C and D in fig. S2) or with only Xenoturbellida (E and F in fig. S1), while for two, deuterostomes were assumed monophyletic (G and H in fig. S1). For all six scenarios, the rest of the tree followed the topology published in (*9*) (figure 1 in the original article). For the last two hypotheses (G and H in fig. S1), we also examined the additional dataset of Cannon *et al.* (Cannon), for which the phylogeny was based on the topology from (*5*) (figure 2 in the original study). As before, we randomly selected 30,000 sites from the original Cannon alignment.

### Branch length estimation: Empirical data

Our first goal was to test whether the site-frequency-heterogeneous (CAT + LG + G) and site-frequency-homogeneous (LG + G) models yield different branch length estimates for either of the two main empirical datasets (i.e., Simion-all and Philippe-all). To achieve this, we used PhyloBayes-MPI version 1.8 (*15*) to estimate a posterior sample of the branch lengths for the two datasets under the LG + G and the CAT + LG + G models (other priors were kept to default values). Each of the four combinations (i.e., Simion-all with LG + G, Simion-all with CAT + LG + G, Philippe-all with LG + G, and Philippe-all with CAT + LG + G) were run twice for 10,000 Markov chain Monte Carlo (MCMC) cycles with a sampling frequency of 1. At 10,000 MCMC cycles, the two runs were assessed for signs of convergence [i.e., effective sample size (ESS) > 100 for each of the runs and for the combined pairs]. The runs that had ESS values lower than 100 were run for 10,000 or 20,000 additional MCMC cycles (table S1).

We also examined the branch length estimates for the different gene sets (i.e., Philippe-best, Philippe-worst, Simion-best, and Simion-worst) under the site-heterogeneous model using the same procedure. In all cases, we used the final 5000 posterior samples of the relevant runs and calculated two sets of specific branches in the two phylogenies: internal branches and the average branch lengths of major clades. We collected the branch length values using a custom Python script available at https://github.com/MaxTelford/XenoCtenoSims. The distributions of all branch length estimates are provided in the form of box plots in Figs. 2 and 4.

#### Internal branches

For the Philippe data, the internal branches measured were those leading to each of the Xenacoelomorpha, Ambulacraria, Xenambulacraria, Chordata, Protostomia, Chordata + Protostomia, Porifera, Bilateria + Cnidaria, Cnidaria, and Bilateria. For the Simion data, internal branches were those leading to each of Cnidaria, Bilateria, Cnidaria + Bilateria, Cnidaria + Bilateria + Placozoa, Ctenophora, Cnidaria + Bilateria + Placozoa + Ctenophora, Porifera, and Metazoa.

#### Average lengths of the major clades

For the Philippe data, we calculated the average distance from each species to the common ancestor for all species of that clade within the following clades: Xenacoelomorpha, Ambulacraria, Chordata, Protostomes, Porifera, Placozoa, and Cnidaria. For the Simion data, we measured the average branch lengths within Bilateria, Cnidaria, Placozoa, Ctenophora, Porifera, and non-Metazoa.

### Branch length estimation: Simulated data

The site-homogeneous and site-heterogeneous models yielded different branch length estimates, and to find which was producing the discrepancy, we performed a test using simulated data. We used two simulated datasets (see the “Simulations” section for details) for which the true tree topology was Xenambulacraria (topology A in fig. S1) and the parameters and branch lengths were estimated on the basis of the Philippe-all dataset. The two datasets differed only in the substitution model used, i.e., for one of them, we assumed a homogeneous site frequency (“Sim-LG + G”) substitution process (i.e., the LG + G model) and for the other, we assumed a heterogeneous process (“Sim-CAT + LG + G”).

For each of our simulated datasets, we next estimated the same branch lengths as before using both heterogeneous and homogeneous models with PhyloBayes using the same procedure as for the empirical data, i.e., we performed four runs: (i) data, Sim-LG + G and model for inference, LG + G; (ii) data, Sim-LG + G and model for inference, CAT + LG + G; (iii) data, Sim-CAT + LG + G and model for inference, LG + G; and (iv) data, Sim-CAT + LG + G and model for inference, CAT-LG + G.

### Model fitting

To determine whether the site-homogeneous (LG + G) or site-heterogeneous (LG + CAT + G) model was a better fit to the Simion-all and Philippe-all datasets, we compared the models using cross-validation (*31*) as implemented in PhyloBayes-MPI. The test was performed in five steps according to the instruction manual: (i) The original 30,000–amino acid alignment for each of the datasets was randomly subsampled to create two subsets, i.e., the training dataset (10,000 sites) and the test dataset (2000 sites). (ii) The parameters of one of the competing models were estimated on the basis of the training dataset by performing 5000 MCMC steps. (iii) Using the estimated model parameters and a given topology (the Porifera-first and the Xenambulacraria, correspondingly), we calculated the likelihood for the test dataset using the “readpb_mpi -cv” option available in PhyloBayes-MPI. (iv) Using the same training and test datasets, the procedure was repeated for the other model. (v) The model yielding the highest likelihood was considered the best fitting. We repeated the test 10 times for each dataset and pair of models, and for all repetitions, the site-heterogeneous model was found to be better than the site-homogeneous model (Δlog*L* = 3036.14 ± 96 for Philippe and Δlog*L* = 3956.02 ± 199 for Simion).

### Simulations

Our next goal was to assess whether the differences in branch length estimates from different models and datasets could result in topological differences. The empirical data alone make this hard to test, as we do not know the true phylogeny. Instead, we simulated data using parameters that match those measured from empirical sequences using the different topological hypotheses and models. All topological hypotheses (A to H) used for the simulations are provided in fig. S1. The simulations were performed using PhyloBayes in two steps:

1) Initially, we estimated the posteriors of branch lengths and model parameters as described above, assuming the following combinations of fixed topology, substitution model, and alignment: (i) topology, A; data, Simion-all; and model, CAT + LG + G; (ii) topology, A; data, Simion-best; and model, CAT + LG + G; (iii) topology, A; data, Simion-worst; and model, CAT + LG + G; (iv) topology, B; data, Simion-all; and model, CAT + LG + G; (v) topology, B; data, Simion-best; and model, CAT + LG + G; (vi) topology, B; data, Simion-worst; and model, CAT + LG + G; (vii) topology, A; data, Simion-all; and model, LG + G; (viii) topology, B; data, Simion-all; and model, LG + G; (ix) topology, C; data, Philippe-all; and model, CAT + LG + G; (x) topology, C; data, Philippe-best; and model, CAT + LG + G; (xi) topology, C; data, Philippe-worst; and model, CAT + LG + G; (xii) topology, D; data, Philippe-all; and model, CAT + LG + G; (xiii) topology, D; data, Philippe-best; and model, CAT + LG + G; (xiv) topology, D; data, Philippe-worst; and model, CAT + LG + G; (xv) topology, C; data, Philippe-all; and model, LG + G; (xvi) topology, D; data, Philippe-all; and model, LG + G; (xvii) topology, E; data, Philippe-all (no acoels); and model, CAT + LG + G; (xviii) topology, E; data, Philippe-best (no acoels); and model, CAT + LG + G; (xix) topology, E; data, Philippe-worst (no acoels); and model, CAT + LG + G; (xx) topology, F; data, Philippe-all (no acoels); and model, CAT + LG + G; (xxi) topology, G; data, Philippe-all; and model, CAT + LG + G; (xxii) topology, H; data, Philippe-all; and model, CAT + LG + G; (xxiii) topology, G; data, Cannon-all; and model, CAT + LG + G; and (xxiv) topology, H; data, Cannon-all; and model, CAT + LG + G. The total number of MCMC cycles required for each combination to reach convergence is provided in table S1.

2) Using the final 5000 posterior samples, we subsampled with a frequency of 1 in 500, which gave us a subset of 100 posterior samples. Using these combinations of branch lengths and model parameters, we simulated data with the “readpb_mpi” tool under the “ppred” option.

For each of the simulated datasets, we inferred the phylogenetic relationships using both a site-frequency-homogeneous and a heterogeneous model (see below) to determine whether using an approximately correct versus a misspecified model results in the recovery of the correct or the conflicting topology in each of the simulated scenarios. Given the large number of simulated datasets (i.e., 2400 in total), it would be challenging to infer all the intended phylogenetic inferences (i.e., 12,000) in a Bayesian context, particularly under the CAT model. As a more practical alternative, we chose a maximum likelihood approach and an approximation of the site-heterogeneous model. We used IQ-TREE version 1.6.11 (*16*) to infer the phylogeny under LG + G (with empirical state frequencies) as the site-frequency-homogeneous model, while for the heterogeneous model, we used the C60 + LG + G + F [Le *et al.* (*17*)]. The C60 model has 60 categories of sites as opposed to the (potentially) infinite sites of the CAT model; however, this simplified model constitutes a good approximation to a full site-heterogeneous model (*17*, *32*) and one that is fast enough (*33*) to process hundreds of simulation replicates. We used the posterior mean site frequency (pmsf) approximation (*33*), as suggested by the IQ-TREE manual, which requires an input tree for the calculation of the weights for each of the frequency vectors, for which we used the phylogeny estimated by the LG + G model.

For a subset of the simulated data, we performed bootstrap analyses to evaluate the strength of support for the inferred topology under both the site-homogeneous and site-heterogeneous model. Specifically, we performed the analyses for the datasets simulated under the Porifera-first and Ctenophora-first hypotheses with the Simion-all dataset as well as under the Xenambulacraria and Nephrozoa hypotheses with the Philippe-all dataset. For each dataset, we performed 1000 ultrafast bootstrap replicates with IQ-TREE (*34*). We did two bootstrap runs: once under the site-homogeneous model and once under the site-heterogeneous model. The analyses were performed with the “-wbt” option that stores the bootstrap trees. Subsequently, using the resulting bootstrap files, we calculated the frequency of occurrence of the conflicting splits for each dataset, i.e., in the Simion-all datasets, we searched for the splits “Ctenophora, Outgroup | Porifera, Remaining Metazoa” and “Porifera, Outgroup | Ctenophora, Remaining Metazoa,” and in the Philippe-all datasets, we searched for the “Xenacoelomorpha, Outgroup | Ambulacraria, Remaining Bilateria” and the “Outgroup, Remaining Bilateria | Xenacoelomorpha, Ambulacraria” splits.

Last, we performed three further tests. First, we tested whether the computationally faster site-heterogeneous model “C10” (with 10 distinct categories of sites) (*17*) would perform similarly to the more complex “C60” (with 60 distinct categories of sites). We used the data simulated under site-heterogeneous models and (i) the Porifera-first and the Ctenophora-first hypotheses (topologies A and B; fig. S1) with the Simion-best, Simion-all, and Simion-worst and (ii) the Xenambulacraria and Nephrozoa hypotheses (topologies C and D; fig. S1) with the Philippe-best, Philippe-all, and Philippe-worst. Second, to assess whether PhyloBayes under the CAT model produces similar results, we used the CAT-LG model to infer the topology of 20 datasets simulated under the Porifera-first, 10 under the Ctenophora-first, 20 under the Xenambulacraria, and 10 under the Nephrozoa topologies. For the Porifera-first and the Xenambulacraria datasets, we selected two sets of simulated data, 10 that recovered the true topology under the C60 model and 10 that recovered the wrong topology (i.e., the Ctenophora-first and the Nephrozoa). Last, we used the CAT-F81 model to infer the phylogeny of 10 datasets simulated under the Ctenophora-first hypothesis and 10 datasets simulated under the Nephrozoa hypothesis. These tests would help us assess whether the simplistic but commonly used Poisson model could cause the erroneous recovery of the Xenambulacraria or Porifera-first topologies. All the datasets used for the CAT inferences (either CAT-LG or CAT-F81) were simulated using parameters learned from the worst data, which were the most challenging ones. The resulting topologies for each set of simulations were summarized into a consensus tree using RaxML (*35*) and are provided in https://github.com/MaxTelford/XenoCtenoSims.

### Composite dataset analyses

To estimate the strength of the bias toward reconstructing the Nephrozoa versus Xenambulacraria topologies, we synthesized datasets with increasing proportions of positions derived from simulations based on the Xenambulacraria tree compared to Nephrozoa. We created 20 pairs of simulated datasets; in each pair, one dataset was simulated under the Xenambulacraria hypothesis and the other under the Nephrozoa. For each pair, we combined the two datasets into a composite alignment with nine different proportions: 10% Xenambulacraria–90% Nephrozoa, 20% Xenambulacraria–80% Nephrozoa, 30% Xenambulacraria–70% Nephrozoa, 40% Xenambulacraria–60% Nephrozoa, 50% Xenambulacraria–50% Nephrozoa, 60% Xenambulacraria–40% Nephrozoa, 70% Xenambulacraria–30% Nephrozoa, 80% Xenambulacraria–20% Nephrozoa, and 90% Xenambulacraria–10% Nephrozoa.

We followed this procedure for two cases: once assuming deuterostomes being monophyletic and once assuming deuterostomes to be paraphyletic. Overall, this gave us 180 composite alignments, and for each of them, we inferred the phylogenetic relationships using IQ-TREE under the site-heterogeneous model C60 + LG + G + F (table S2). The inference was performed with the pmsf approximation as described earlier.

## Supplementary Material

http://advances.sciencemag.org/cgi/content/full/6/50/eabc5162/DC1

Adobe PDF - abc5162_SM.pdf

Topology-dependent asymmetry in systematic errors affects phylogenetic placement of Ctenophora and Xenacoelomorpha

## SUPPLEMENTARY MATERIALS

Supplementary material for this article is available at http://advances.sciencemag.org/cgi/content/full/6/50/eabc5162/DC1

## Appendix

View/request a protocol for this paper from *Bio-protocol*.
