# Supplementary material for: Topology-dependent asymmetry in systematic errors affects phylogenetic placement of Ctenophora and Xenacoelomorpha
Source: Sci Adv. 2020 Dec 11;6(50):eabc5162. doi: 10.1126/sciadv.abc5162 (PMC7732190; doi:10.1126/sciadv.abc5162)
Supplement: http://advances.sciencemag.org/cgi/content/full/6/50/eabc5162/DC1 [file supp_6_50_eabc5162__index.html]

Science Advances | Science AdvancesAAASSearchScience AdvancesMenu

## Supplementary Materials

# Topology-dependent asymmetry in systematic errors affects phylogenetic placement of Ctenophora and Xenacoelomorpha

Paschalia Kapli and Maximilian J. Telford

Download Supplement

**This PDF file includes:**

- Fig. S1
- Tables S1 and S2

**Files in this Data Supplement:**

- Adobe PDF - abc5162\_SM.pdf
