# Supplementary material for: Topology-dependent asymmetry in systematic errors affects phylogenetic placement of Ctenophora and Xenacoelomorpha
Source: Sci Adv. 2020 Dec 11;6(50):eabc5162. doi: 10.1126/sciadv.abc5162 (PMC7732190; doi:10.1126/sciadv.abc5162)
Supplement: http://advances.sciencemag.org/cgi/content/full/6/50/eabc5162/DC1 [file supp_6_50_eabc5162__1.pdf]

Science Advances
AAAS

advances.sciencemag.org/cgi/content/full/6/50/eabc5162/DC1

Supplementary Materials for

Topology-dependent asymmetry in systematic errors affects phylogenetic placement of Ctenophora and Xenacoelomorpha

Paschalia Kapli and Maximilian J. Telford*

*Corresponding author. Email: m.telford@ucl.ac.uk

Published 11 December 2020, *Sci. Adv.* **6**, eabc5162 (2020)
DOI: 10.1126/sciadv.abc5162

This PDF file includes:

Fig. S1
Tables S1 and S2

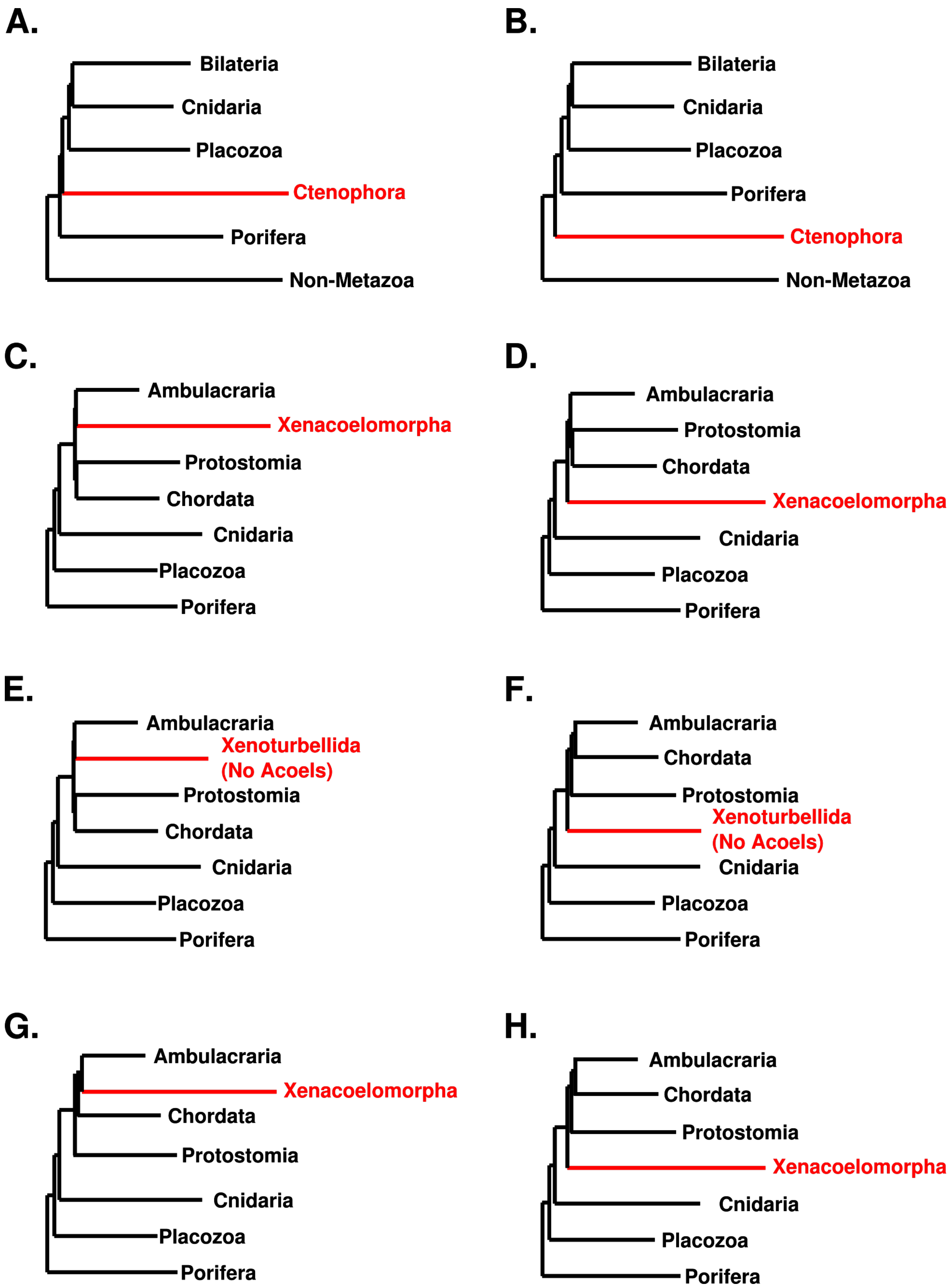

Fig. S1. Summary topologies used as guide trees in the simulations. A and B. Alternative trees relating Ctenophora. C and D. Alternative trees relating Xenacoelomorpha. E and F. Alternative trees relating *Xenoturbella* (long branched Acoelomorpha removed). G and H. Alternative trees relating Xenacoelomorpha with Deuterostomia constrained to be monophyletic.

Table S1. Summary of tree topologies reconstructed on different simulated data sets and using different models. Each row refers to a different set of simulation conditions that are described in the first two columns, i.e., the topology that was used as the guide tree (additional explanations are given at the bottom of the table) and specific alignment (in parenthesis is given the reference to the original study that published the data). In the next three columns are provided the model, the number of MCMC steps and the number of independent runs performed with Phylobayes for inferring the posterior sample of parameters used for the simulations. In the remaining columns are shown the percentages of recovering the conflicting topologies (Porifera-first or Xenambulacraria vs. Ctenophora first or Nephrozoa) when the topology was inferred with different models (LG, C10-LG, C60-LG, CAT-LG, CAT-F81). In most cases the percentages of the two competing topologies sum up to 100 in the cases that they don't an explanation is provided in the "Comments" column. A "-" is shown for the instances where the analyses were not performed.

	Topology	Alignment	Model	MCMC Cycles	Runs	Porifera-first / Xenambulacraria	Ctenophora-first / Nephrozoa	Porifera-first / Xenambulacraria	Ctenophora-first / Nephrozoa	Porifera-first / Xenambulacraria	Ctenophora-first / Nephrozoa	Porifera-first / Xenambulacraria	Ctenophora-first / Nephrozoa	Comments
**1**	Porifera-first (A)[*]	Simion-Best (*8*)	CAT+LG+G	10K	2	7	93	51	49	89	11	-	-	
**2**	Porifera-first (A)[*]	Simion-All (*8*)	CAT+LG+G	10K	2	2	98	43	57	88	12	-	-	
**3**	Porifera-first (A)[*]	Simion-Worst (*8*)	CAT+LG+G	10K	2	0	100	22	78	65	35	20	0	
**4**	Ctenophora-first (B)[†]	Simion-Best (*8*)	CAT+LG+G	10K	2	0	100	0	100	0	100	-	-	
**5**	Ctenophora-first (B)[†]	Simion-All (*8*)	CAT+LG+G	10K	2	0	100	0	100	0	100	-	-	
**6**	Ctenophora-first (B)[†]	Simion-Worst (*8*)	CAT+LG+G	10K	2	0	100	0	100	0	100	CAT-LG: 0 , CAT-F81: 0	CAT-LG: 10 , CAT-F81: 10	
**7**	Porifera-first (A)[*]	Simion-All (*8*)	LG+G	10K	2	100	0	-	-	100	0	-	-	
**8**	Ctenophora-first (B)[†]	Simion-All (*8*)	LG+G	10K	2	0	100	-	-	0	100	-	-	
**9**	Xenambulacraria (C)[‡]	Philippe-Best (*9*)	CAT+LG+G	20K	2	26	74	91	9	100	0	-	-	
**10**	Xenambulacraria (C)[‡]	Philippe-All (*9*)	CAT+LG+G	20K	2	10	90	69	31	95	5	-	-	
**11**	Xenambulacraria (C)[‡]	Philippe-Worst (*9*)	CAT+LG+G	10K	2	0	100	1	99	25	73	18	0	C60, CAT : in 2 replicates Xenacoelomorpha sister to Chordata-Protostomia.
**12**	Nephrozoa (D)[§]	Philippe-Best (*9*)	CAT+LG+G	20K	2	0	100	0	100	0	100	-	-	
**13**	Nephrozoa (D)[§]	Philippe-All (*9*)	CAT+LG+G	10K	2	0	100	0	100	0	100	-	-	
**14**	Nephrozoa (D)[§]	Philippe-Worst (*9*)	CAT+LG+G	10K	2	0	100	0	100	0	100	CAT-LG: 0 , CAT-F81: 0	CAT-LG: 10 , CAT-F81: 10	
**15**	Xenambulacraria (C)[‡]	Philippe-All (*9*)	LG+G	10K	2	100	0	-	-	100	0	-	-	
**16**	Nephrozoa (D)[§]	Philippe-All (*9*)	LG+G	10K	2	0	100	-	-	0	100	-	-	
**17**	Xenambulacraria (E)[‖]	Philippe-Best (*9*)	CAT+LG+G	20K	2	92	8	-	-	100	0	-	-	
**18**	Xenambulacraria (E)[‖]	Philippe-All (*9*)	CAT+LG+G	20K	2	52	48	-	-	89	9	-	-	C60: in 2 replicates Xenacoelomorpha sister to Chordata-Protostomia.
**19**	Xenambulacraria (E)[‖]	Philippe-Worst (*9*)	CAT+LG+G	20K	2	43	56	-	-	82	13	-	-	C60: in 5 replicates, LG: in 1 replicate Xenacoelomorpha is sister to Chordata-Protostomia
**20**	Nephrozoa (F)[¶]	Philippe-All (*9*)	CAT+LG+G	10K	2	0	100	-	-	0	100	-	-	
**21**	Xenambulacraria (G)[#]	Philippe-All (*9*)	CAT+LG+G	30K	2	54	46	-	-	100	0	-	-	
**22**	Nephrozoa (H)[**]	Philippe-All (*9*)	CAT+LG+G	30K	2	0	100	-	-	0	100	-	-	
**23**	Xenambulacraria (G)[#]	Cannon (*5*)	CAT+LG+G	20K	2	91	8	-	-	99	1	-	-	LG: in 1 replicate Xenacoelomorpha sister to Deuterostomia
**24**	Nephrozoa (H)[**]	Cannon (*5*)	CAT+LG+G	20K	2	0	100	-	-	0	100	-	-	

[*]Topology A. Full taxon sampling of the Simion datasets with Porifera sister to all other animals
[†]Topology B. Full taxon sampling of the Simion datasets with Ctenophora sister to all other animals
[‡]Topology C. Full taxon sampling of the Philippe datasets with Xenacoelomorpha sister to Ambulacraria and Deuterostomes paraphyletic
[§]Topology D. Full taxon sampling of the Philippe datasets with Xenacoelomorpha sister to Nephrozoa and Deuterostomes paraphyletic
[‖]Topology E. Same as C but without Acoelomorpha
[¶]Topology F. Same as D but without Acoelomorpha
[#]Topology G. Full taxon sampling of the Philippe or Cannon datasets with Xenacoelomorpha sister to Ambulacraria and Deuterostomes monophyletic
[**]Topology H. Full taxon sampling of the Philippe or Cannon datasets with Xenacoelomorpha sister to Nephrozoa and Deuterostomes monophyletic

Table S2. Summary of results for composite datasets analyses. The composite datasets were created with different proportions of simulated data under the two conflicting topologies. The proportions are given in the first two columns of the two tables. For each combination we created 20 datasets. The inferred topology under the site-heterogeneous model C60 was either the Xenambulacraria or the Nephrozoa, the exact frequencies are given in the third and fourth columns of the two tables. The analyses were repeated for two conditions affecting the relationships of the Deuterostome clades (i.e., Chordata and Ambulacraria), either forming a paraphyletic clade (top) or a monophyletic clade (bottom).

	Topology of simulated dataset (paraphyletic Deuterostomia)		Inferred topology	
	Xenambulacraria	Nephrozoa,	Xenambulacraria	Nephrozoa,
Percentage	10%	90%	0%	100%
	20%	80%	0%	100%
	30%	70%	0%	100%
	40%	60%	0%	100%
	50%	50%	0%	100%
	60%	40%	30%	70%
	70%	30%	30%	70%
	80%	20%	50%	50%
	90%	10%	90%	10%

	Topology of simulated dataset (monophyletic Deuterostomia)		Inferred topology	
	Xenambulacraria	Nephrozoa,	Xenambulacraria	Nephrozoa,
Percentage	10%	90%	0%	100%
	20%	80%	0%	100%
	30%	70%	0%	100%
	40%	60%	0%	100%
	50%	50%	0%	100%
	60%	40%	0%	100%
	70%	30%	20%	80%
	80%	20%	60%	30%
	90%	10%	100%	0%
